# The Effect of Caffeine on Movement-Related Cortical Potential Morphology and Detection

**DOI:** 10.3390/s24124030

**Published:** 2024-06-20

**Authors:** Mads Jochumsen, Emma Rahbek Lavesen, Anne Bruun Griem, Caroline Falkenberg-Andersen, Sofie Kirstine Gedsø Jensen

**Affiliations:** Department of Health Science and Technology, Aalborg University, 9220 Aalborg, Denmark

**Keywords:** movement-related cortical potential, brain-computer interface, movement intention, EEG, caffeine, coffee, bereitschaftspotential, contingent negative variation

## Abstract

Movement-related cortical potential (MRCP) is observed in EEG recordings prior to a voluntary movement. It has been used for e.g., quantifying motor learning and for brain-computer interfacing (BCIs). The MRCP amplitude is affected by various factors, but the effect of caffeine is underexplored. The aim of this study was to investigate if a cup of coffee with 85 mg caffeine modulated the MRCP amplitude and the classification of MRCPs versus idle activity, which estimates BCI performance. Twenty-six healthy participants performed 2 × 100 ankle dorsiflexion separated by a 10-min break before a cup of coffee was consumed, followed by another 100 movements. EEG was recorded during the movements and divided into epochs, which were averaged to extract three average MRCPs that were compared. Also, idle activity epochs were extracted. Features were extracted from the epochs and classified using random forest analysis. The MRCP amplitude did not change after consuming caffeine. There was a slight increase of two percentage points in the classification accuracy after consuming caffeine. In conclusion, a cup of coffee with 85 mg caffeine does not affect the MRCP amplitude, and improves MRCP-based BCI performance slightly. The findings suggest that drinking coffee is only a minor confounder in MRCP-related studies.

## 1. Introduction

The movement-related cortical potential (MRCP) is a brain potential observed in electroencephalographic (EEG) recordings as a negative potential associated with a voluntarily produced movement [1]. Moreover, the MRCP is elicited by imagined movements [2,3,4] and by attempted movements in patients with motor impairments such as stroke [2,3], cerebral palsy [5], spinal cord injury [6,7,8], and amyotrophic lateral sclerosis [9,10,11]. The MRCP can be considered a general term describing self-paced and cue-based/externally paced movements which are known as bereitschaftspotential [12] and contingent negative variation [13], respectively. The MRCP can be divided into different components with the readiness potential (RP) starting ~2 s prior to the movement onset until ~0.5 s prior the movement onset; this potential is seen as a slight increase in negativity. The RP is followed by the negative slope (NS), which is seen as a steeper increase in negativity until ~0.1 s before the movement onset. The NS is followed by the motor potential (MP), which is observed close to the movement onset. After the movement onset, the movement-monitoring potential is observed, which lasts ~1 s where the negative brain potential returns to the baseline. It should be noted that different names and definitions of the components have been used in the literature [14,15,16]. Common to all MRCPs associated with different movement types is a general activation pattern involving the brain areas associated with movement planning and execution along with a major contribution from the supplementary motor area, premotor cortex, and primary motor cortex [14]. However, self-paced movements have been associated with higher activity in the supplementary motor area compared to externally paced movements, which show higher activity in the dorsal premotor cortex [17]. The MRCP has been reported to be elicited during a variety of different movement types (reviewed in [1]) with the upper extremities, lower extremities, and the tongue [18]. However, there are several factors affecting the morphology of the MRCP. Factors such as perceived effort [19], movement type [20,21,22], movement kinetics and trajectories [23,24], and attention variations modulate the amplitude of one or more of the components of the MRCP [25]. Several studies have reported how these factors affect the MRCP morphology and contribute to the knowledge regarding factors to control for in applications where the MRCP is used. These applications include the MRCP as a tool for studying motor control [14,26], disease mechanisms [27], cortical excitability [28,29,30,31], and motor skill learning [32,33]. Another major application of the MRCP is for brain–computer interface (BCI) control where it can be used as a control signal to control external technology, especially for neurorehabilitation where Hebbian-associated plasticity is induced [34,35,36,37,38]. In this application, the MRCP, as a control signal, is useful since it activates the areas of the brain associated with movement, and it is possible to predict movements and attempted movements in patients with motor impairments [2,3,39,40], which is important to adhere to the strict temporal association between motor cortical activation and inflow of relevant somatosensory feedback from, e.g., electrical nerve stimulation [37]. As previously mentioned, several factors affect the MRCP morphology and hence the application where the MRCP is used. One factor, however, that has not been studied in detail is the effect of caffeine on the MRCP morphology. Caffeine has generally been attributed to increases in subjective energy and arousal, general improvement of information processing, and modulating attention (see [41] for a review about caffeine and brain function). This has been studied using different brain imaging techniques including EEG through event-related potentials. The MRCP is also considered an event-related potential; however, the evidence of the effect of caffeine on the MRCP is limited to a few studies with mixed results. A slight amplitude increase of 1–2 µV/s of the contingent negative variation was reported after consuming 300 mg caffeine mixed with 200 mL hot water [42]. In another study, it was found that caffeine increased the amplitude of the bereitschaftspotential of tasks associated with lower levels of exhaustion such that they were similar to amplitudes associated with tasks of submaximal activation without caffeine [43]. A reduction of the amplitude of the contingent negative variation [44] and MRCP has been reported as well after consuming caffeine, which was attributed to increased spinal and supraspinal excitability [45]. Since the MRCP amplitude may be affected by caffeine, it can also affect the performance of an MRCP-based BCI since the signal-to-noise ratio is a determinant of BCI performance. Therefore, the aim of this study is twofold: (1) to investigate if a cup of coffee with 85 mg caffeine affects the pre-movement components of the MRCP, and (2) to investigate the effect of a cup of coffee on single-trial MRCP classification, which serves as an estimate of BCI performance.

## 2. Materials and Methods

### 2.1. Participants

Twenty-six healthy participants were recruited (14 females and 12 males with a mean age of 25 ± 10 years). All participants provided their written informed consent. The procedures were approved by The North Denmark Region Committee on Health Research Ethics (approval number: N-20230015).

### 2.2. Experimental Setup

**Figure 1:**
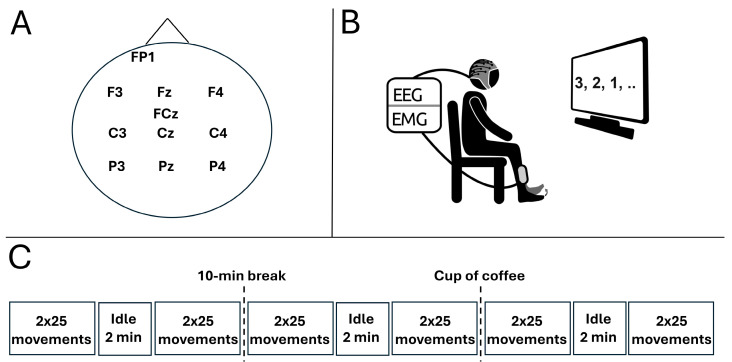
(**A**) Channel locations of the recorded EEG channels. (**B**) Continuous EEG and EMG were recorded, while visually cued ankle dorsiflexions were performed. (**C**) Overview of the progression of the experiment.

The participant was seated in a comfortable chair facing a computer monitor one meter away from the participant (see Figure 1) in a brightly lit and quite room. An EEG cap was mounted, and the participant was instructed about the experimental procedure. The participant executed 100 dorsiflexions of the right ankle joint in three separate sessions. Each session was separated by a 10-min break. In the 10-min break after the second session, the participant consumed a cup of coffee (150 mL) containing approximately 85 mg caffeine. Two sessions, each containing 100 movements, preceded the intake of caffeine followed by another session with 100 movements. The dose of caffeine was chosen to reflect a moderate intake. The two sessions prior to the intake of caffeine served as a reference period to determine the changes in MRCP morphology and single-trial MRCP detection without any caffeine intake. In each session, 4 × 25 movements were performed with a 2-min break in between each block of 25 movements. A ballistic movement with a low level of force was performed every 10 s, and the participant was instructed to sit as still as possible and avoid blinking and activating facial muscles three seconds prior to the movement onset until two seconds after. The timing of the movements was guided by a counter. Prior to the first session, the participant spent approximately five minutes familiarizing themselves with the task and experimental setup. In each session after the first 50 movements, a 2-min recording of idle activity with open eyes was obtained.

### 2.3. Recordings

#### 2.3.1. EEG

Eleven channels of continuous EEG were recorded from FP1, F3, Fz, F4, FCz, C3, Cz, C4, P3, Pz, and P4 according to the International 10–20 System (see Figure 1A). The EEG was acquired using passive sintered Ag/AgCl ring electrodes (EASYCAP GmbH, Wörthsee, Germany) positioned in a flexible cap (EASYCAP GmbH, Wörthsee, Germany) and sampled with 500 Hz (EEG amplifiers, Nuamps Express, Compumedics Neuroscan, Freiberg, Germany). All channels were referenced to the right mastoid bone and grounded on the forehead. Throughout the experiment, the impedance was below 10 kΩ.

#### 2.3.2. EMG

Using the same amplifier as for the EEG recordings, EMG was recorded in a bipolar derivation (Ambu^®^ Neuroline 720 Surface Electrodes, Ballerup, Denmark) from the right tibialis anterior muscle. Hence, the EEG was synchronized to the EMG. From the EMG recordings, movements were identified, and the EEG was divided into epochs based on this information.

### 2.4. Data Analysis—EMG Detection

**Figure 2:**
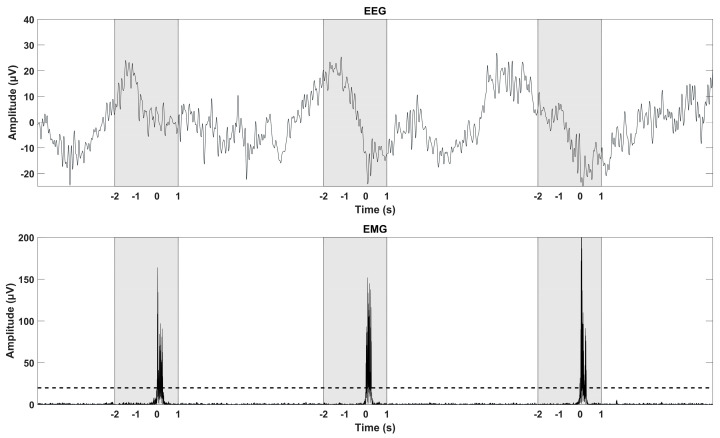
The continuous EEG (**top**) and EMG (**bottom**) are displayed for three movements. The dashed black line in the EMG plot shows the participant-specific threshold. The shaded areas indicate the extracted epochs.

The continuous EMG was bandpass filtered with a fourth order zero-phase shift Butterworth filter between 20 and 40 Hz to remove noise and rectified to identify the movements from a clear EMG with suppressed electrical baseline activity. The movements were identified using a participant-specific detection threshold (see Figure 2). All identified movements were manually checked. The identified movements were used to divide the continuous EEG into epochs. In the following, the EMG-identified movements will be referred to as movement onsets. 

### 2.5. Data Analysis—MRCP Morphology

The continuous EEG was bandpass filtered with a fourth order zero-phase shift Butterworth filter between 0.1 and 10 Hz to reduce the effect of noise on the MRCP and baseline drift. The EEG was divided into three-second epochs, two seconds prior until one second after the movement onset (see Figure 2). Epochs containing amplitudes exceeding ±125 µV were rejected from further analysis to account for artifacts not originating from the brain activity. The remaining epochs were averaged such that three average MRCPs were obtained for session 1, 2, and 3, respectively. The amplitudes of the RP, NS, and MP were extracted. The amplitudes of the RP, NS, and MP were defined as follows: Mean amplitude from −2 to −0.5 s prior the movement onset (RP), mean amplitude from −0.5 to −0.1 s prior the movement onset (NS), and mean amplitude from −0.1 to 0.1 s with respect to the movement onset (MP) [15]. The RP, NS, and MP were extracted from Fz, FCz, and Cz. Morphological analysis was performed for Fz, FCz, and Cz. 

### 2.6. Data Analysis—MRCP Detection

#### 2.6.1. Pre-Processing

The continuous EEG was bandpass filtered with a fourth order zero-phase shift Butterworth filter between 0.1 and 30 Hz to reduce the effect of noise on the MRCP and baseline drift. The cutoff frequency at 30 Hz was chosen to include the movement-related information in the mu and beta rhythm frequency range. Afterwards, a large Laplacian filter was applied with Cz as the center channel based on the following formula to obtain a surrogate channel:

Surrogate channel = Cz − (F3 + Fz + F4 + C3 + C4 + P3 + Pz + P4)/8(1)

The surrogate channel was used in the remaining detection analysis. The continuous EEG in the surrogate channel was divided into two-second epochs, two seconds prior until the movement onset (see Figure 2). Epochs containing amplitudes exceeding ±125 µV were rejected from further analysis. The same pre-processing was performed for the recordings of idle activity. From these recordings, random two-second epochs were extracted such that the same number of MRCP and idle epochs was used for the classification analysis. 

#### 2.6.2. Feature Extraction and Classification

From each epoch of the surrogate channel, three feature types were extracted: Temporal, spectral, and template-matching features. The temporal features were the mean amplitudes from −2 to −1.5, −1.5 to −1, −1 to −0.5, and −0.5 to 0 s with respect to the movement onset. Also, the difference in amplitude between the first (−2 to −1 s) and second (−1 to 0 s) half of the epoch was used as feature. The spectral features consisted of estimated power spectral density in 1-Hz bins from 8 to 30 Hz. A 1-s Hamming window with 0.5 s overlap was used to estimate the power spectral density. A total of 23 spectral features were extracted. A single template-matching feature was calculated. The average across all epochs of the training data was used as a template, and the cross-correlation between each epoch and the template (MRCP and idle activity) was calculated with zero time lag. This value was used as a feature. 

The features were classified using three different classifiers: (1) a random forest classifier with 512 trees, (2) linear discriminant analysis, and (3) k-nearest neighbors with k = 5. Leave-one-out cross-validation was used for dividing the data into a training set and a testing set. It was a two-class classification scenario with the MRCP and idle activity as the two classes. This classification was performed on each participant individually in session 1, session 2, and session 3. This served as an estimate of MRCP-based BCI performance. 

### 2.7. Statistical Analysis

A one-way repeated measures analysis of variance (ANOVA) test was performed for each component (RP, NS, and MP) for Fz, FCz, and Cz, with session as the factor (three levels: Pre1, Pre2, and Post with respect to caffeine intake); i.e., nine tests were performed with the amplitude of the respective MRCP component as dependent variable. Moreover, a two-way repeated measures ANOVA with session (three levels: Pre1, Pre2, and Post with respect to caffeine intake) and classifier (three levels: Random forest, linear discriminant analysis, and k-nearest neighbors) as factors was performed on the classification accuracies. The Greenhouse–Geisser correction was applied if the assumption of sphericity was violated. Significant test statistics of the ANOVA tests were followed up with a Bonferroni post hoc test to account for multiple comparisons. All tests were considered significant when *p* < 0.05. 

## 3. Results

In the pre-processing step of the analysis, 4.1 ± 8.6, 0.4 ± 0.9, and 0.6 ± 1.2 epochs were rejected from further analysis for session one (prior caffeine intake), session two (prior caffeine intake), and session three (after caffeine intake), respectively.

### 3.1. MRCP Morphology

**Figure 3:**
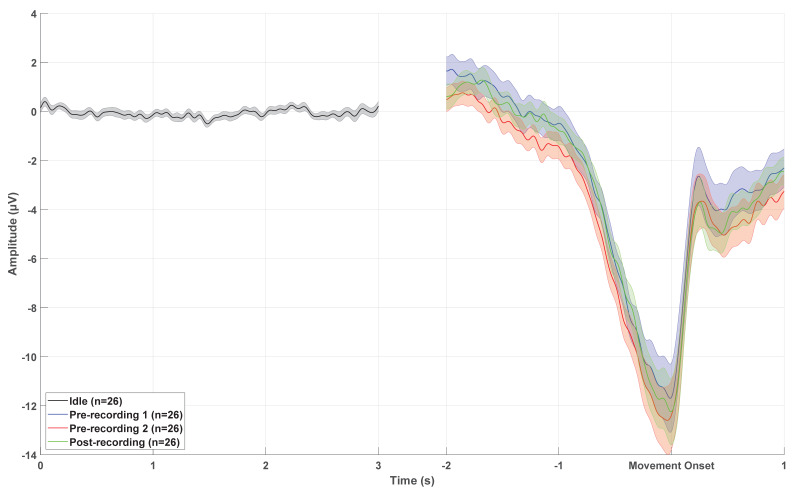
Grand average plot of idle brain activity, the two recording sessions prior to caffeine intake, and the recording session post caffeine intake. The solid lines indicate the mean across the participants, and the shaded area is the standard error across the participants.

The results of the morphological analysis are summarized in Table 1 and Figure 3. A clear increase in negativity was observed from the RP until MP. Generally, the amplitudes of the MRCP components were slightly higher for the Pre-two session compared to the Pre-one and Post session. The statistical analysis for RP revealed a significant difference in amplitude between sessions for FCz (F_(2,50)_ = 3.46; *p* = 0.04; η^2^ = 0.12) and Cz (F_(2,50)_ = 3.69; *p* = 0.03; η^2^ = 0.13). The post hoc analysis revealed a higher amplitude for the Pre2-session compared to the Post-session for Cz, but no difference between sessions was observed for FCz after applying the Bonferroni correction. There was no difference in the RP amplitude for Fz across the three sessions (F_(2,50)_ = 1.54; *p* = 0.22; η^2^ = 0.06). 

The statistical analysis for NS revealed a significant difference in amplitude between sessions for FCz (F_(1.6,40.5)_ = 3.91; *p* = 0.04; η^2^ = 0.14). The post hoc analysis revealed a higher amplitude for the Pre-two session compared to the Pre-one session. There was no difference in the NS amplitude for Fz (F_(1.3,33.4)_ = 2.29; *p* = 0.13; η^2^ = 0.08) and Cz (F_(1.4,33.9)_ = 2.38; *p* = 0.12; η^2^ = 0.09) across the three sessions.

The statistical analysis for MP revealed a significant difference in amplitude between sessions for FCz (F_(1.5,37.2)_ = 4.94; *p* = 0.02; η^2^ = 0.17). The post hoc analysis revealed a higher amplitude for the Pre-two session compared to the Pre-one session. There was no difference in the MP amplitude for Fz (F_(1.5,38.4)_ = 1.13; *p* = 0.32; η^2^ = 0.04) and Cz (F_(1.5,36.8)_ = 2.14; *p* = 0.14; η^2^ = 0.08) across the three sessions. 

### 3.2. MRCP Detection

The results of the classification analysis for estimating the MRCP detection are presented in Table 2. There was an increase in the classification accuracy from the pre-sessions to the post-session for the random forest and linear discriminant analysis classifiers. The statistical analysis revealed a significant effect of session (F_(2,50)_ = 3.84; *p* = 0.03; η^2^ = 0.13), with an increase in classification accuracy from the first pre-session to the post-session. Moreover, a significant effect of classifier was found (F_(1.3,33.7)_ = 45.47; *p* < 0.001; η^2^ = 0.65), with higher classification accuracies for random forest and linear discriminant analysis compared to k-nearest neighbors. There was no interaction between session and classifier (F_(4,100)_ = 1.92; *p* = 0.11; η^2^ = 0.07). It should be noted that there was a wide span of classification accuracies across participants. 

## 4. Discussion

The largest difference in amplitudes was between the two recordings prior to caffeine consumption. The two measurements were performed to establish the natural variation when performing two recordings without any intervention. Thus, the results show that the difference between recordings prior to caffeine consumption exceeds the effect that caffeine may have. This could be attributed to differences in attention [25], or changes in mental state. It has previously been shown that placebo caffeine can modulate fatigue and the MRCP amplitude [46]. Caffeine has previously been reported to affect the amplitude of the MRCP, but mixed results have been reported and generally with small changes in amplitude [42,43,44,45]. In the current study, a lower dose of caffeine was consumed (85 mg vs. 150–300 mg) compared to the previous studies, which suggests that the effect of a cup of coffee with 85 mg caffeine would have a less modulatory effect compared to higher doses of caffeine. In future studies, the effect of multiple doses of caffeine could be investigated, as well as the measurement period after caffeine intake to investigate the evolution of the modulatory effect. In future work, a relative dose of caffeine should be used to account for difference in body weight across participants. This is a limitation of the current study, but 85 mg caffeine is a moderate dose, and because the study followed a repeated measures design, it is not expected to affect the findings. Also, it could be relevant to include information about caffeine consumption habits and sleep quality in the analysis. Caffeine has been reported to normalize the amplitude of event-related potentials in sleep-deprived participants, but with a dose of 400 mg of caffeine [47]. 

The MRCP detection, which is an estimate of MRCP-based BCI performance, improved slightly by two percentage points after caffeine consumption, but similar performance was expected due to the MRCP morphology, which was similar across the three recording sessions. Previous studies have shown that coffee consumption improves the performance of a P300-based BCI slightly [48], and that abstinence from caffeine reduces the P300-amplitude [49], which would affect BCI performance negatively. The effect of caffeine on motor-imagery-based BCI performance has been investigated as well [50], where no changes in performance were observed. The results of the current study and existing literature suggest that the effect of caffeine on BCI performance is limited. However, the findings in the current study need to be validated with an online MRCP-based BCI since offline analysis was performed where epochs were extracted with a priori knowledge of when the movement occurred, and hence perfect alignment between the MRCP and movement onset was obtained. Thus, the estimated BCI performance is expected to be an optimistic estimation, and a reduction in actual BCI control is expected. Online BCI performance could be affected by caffeine to some degree if the user becomes more focused or attentive (as reviewed in [41]) so that artifacts can be avoided that may cause false positive detections or a false negative by e.g., blinking while trying to activate the BCI. In this scenario, the MRCP could be affected by the blink artefact. Drifts in attention have been shown to decrease MRCP amplitudes [25], which reduces the signal-to-noise ratio and hence the BCI performance. The behavioral effect of caffeine on attention could potentially help maintain BCI performance if the user’s attention starts to drift, but this needs to be tested. Caffeine has also been reported to affect fatigue [41], which could indicate that it will not affect BCI performance negatively. BCI performance for mental imagery has been reported to be reduced by fatigue [51], although evidence from motor imagery BCI performance suggests that the performance is not significantly impeded by fatigue [52].

## 5. Conclusions

A cup of coffee with a moderate amount of caffeine (85 mg) does not affect the amplitude of the pre-movement components of the MRCP. Moreover, the MRCP detection shows slightly higher classification accuracies after the intake of caffeine. In future studies, the effect of caffeine could be investigated for online MRCP-based BCI performance. 

## Figures and Tables

**Table 1:** Overview of the components of the movement-related cortical potential for the two sessions prior to caffeine intake (Pre 1 and Pre 2) and the session after caffeine intake (Post). All values are presented as mean ± standard error. RP: Readiness potential. NS: Negative slope. MP: Motor potential.

Channel	Component	Pre 1 (µV)	Pre 2 (µV)	Post (µV)
Fz	RP	−1.4 ± 0.4	−2.0 ± 0.4	−1.5 ± 0.4
	NS	−6.5 ± 0.7	−7.2 ± 0.6	−6.2 ± 0.6
	MP	−7.9 ± 0.8	−8.6 ± 0.8	−8.2 ± 0.8
FCz	RP	−0.5 ± 0.5	−1.3 ± 0.4	−0.8 ± 0.4
	NS	−8.2 ± 0.7	−9.5 ± 0.7	−8.6 ± 0.7
	MP	−10.3 ± 1.0	−11.6 ± 1.0	−11.2 ± 0.9
Cz	RP	−0.5 ± 0.6	−1.4 ± 0.4	−0.7 ± 0.4
	NS	−9.0 ± 0.9	−10.0 ± 0.8	−9.1 ± 0.9
	MP	−10.8 ± 1.3	−11.8 ± 1.3	−11.5 ± 1.3

**Table 2:** Overview of the detection of movement-related cortical potentials. The mean and standard error are calculated across the participants.

Classifier		Pre 1 (%)	Pre 2 (%)	Post (%)
Random forest	Mean ± standard error	79.3 ± 1.5	80.1 ± 1.9	82.3 ± 1.8
	Range [min–max]	[65.0–94.5]	[61.5–96.0]	[55.0–98.0]
Linear discriminant analysis	Mean ± standard error	78.8 ± 1.9	81.0 ± 2.0	82.4 ± 1.7
	Range [min max]	[62.5–97.0]	[58.0–98.0]	[63.5–99.0]
K-nearest neighbors	Mean ± standard error	71.3 ± 2.1	73.6 ± 2.4	72.8 ± 2.5
	Range [min max]	[51.6–94.0]	[55.5–96.0]	[50.5–96.0]

## Data Availability

The data that support the findings of this study are available upon reasonable request from the corresponding author.
